# Genome resource of *Phlyctema vagabunda* strain 19EL15, a pathogen of post-harvest bull’s eye rot of apple

**DOI:** 10.1128/mra.01220-24

**Published:** 2025-09-23

**Authors:** Greice Amaral Carneiro, Sanja Baric, Selena Tomada, Chiara Fiorenzani, Riccardo Baroncelli, Andreas Bühlmann

**Affiliations:** 1 Department of Agricultural and Food Sciences (DISTAL), University of Bologna 9296, Bologna, Italy; 2 Faculty of Agricultural, Environmental and Food Sciences, Free University of Bozen-Bolzano 18956, Bozen-Bolzano, Italy; 3 Competence Centre for Plant Health, Free University of Bozen-Bolzano 18956, Bozen-Bolzano, Italy; 4 Regional Agency for Rural Development (ERSA) 315073 , Pozzuolo del Friuli, Italy; 5 Agroscope, Research Division Food Microbial Systems, Wädenswil, Switzerland; Indiana University, Bloomington, Bloomington, Indiana, USA

**Keywords:** long-read sequencing, genome annotation, genome assembly, fungal genome, RNAseq, short-read sequencing, Sabouraud agar, apple juice agar, apple leaf extract agar, PromethION device

## Abstract

*Phlyctema vagabunda* is a plant pathogenic fungus widespread in Europe and North America that causes severe damages to different crop species. This announcement reports the genome sequence of *P. vagabunda* 19EL15 strain, associated with bull’s eye rot of apple fruit, paving the way for future biological research.

## ANNOUNCEMENT

The fungus *Phlyctema vagabunda* Desm. causes bull’s eye rot of apple and pear, resulting in significant post-harvest losses (1, 2). Prevalent in Europe and North America, this pathogen is also associated with leprosy of olive trees (3, 4).

The isolate 19-DSS-BS-EL-2-015 (short 19EL15) of *P. vagabunda* was obtained in 2019 from an apple fruit with bull’s eye rot in South Tyrol (northern Italy) (1). The monoconidial strain was cultured on potato dextrose agar (PDA) covered with cellophane at 20°C for 7 days. Genomic DNA for short-read sequencing was extracted from mycelium using DNeasy Plant Mini Kit (Qiagen, Hilden, Germany). For long-read sequencing, DNA was purified from spores via an adapted protocol (5) without shearing, which included freeze-drying, liquid nitrogen grinding, lyticase digestion (Sigma-Aldrich, St. Louis, MO, USA), and SDS lysis buffer treatment.

Paired-end reads (2 × 300 bp, *n* = 1,569,489) were generated with the Illumina MiSeq System using Nextera XT DNA Library Preparation Kit and MiSeq Reagent Kit v3 (600 cycle) (Illumina, San Diego, CA, USA). Illumina MiSeq software handled demultiplexing and adapter trimming, and quality was verified with FastQC v0.11.9 (6). Long-read sequencing was facilitated by preparing the library with a bead-free protocol (7) with the ligation sequencing kit (SQK-LSK110), replacing the SRE XL with 9% PEG/NaCl and on a FLO-PRO002 flow cell using the PromethION device from Oxford Nanopore Technologies (ONT, UK) controlled via MinKNOW v21.11.7. Base calling and demultiplexing were managed by Guppy v5.1.13 (ONT), followed by adapter removal using Porechop v0.2.3 (8), incorporating an additional step to eliminate internal adapters based on a 90% identity threshold. ONT reads (*n* = 2,126,159, N50 = 17,085 bp) were assembled with Flye v2.9 (9), followed by three polishing iterations and further refinement using short reads with Pilon v1.24 (10). Assembly statistics (Table 1) were assessed with QUAST v5.2.0 (11), yielding 13 scaffolds and a total genome size of 38.34 Mb. The N50 statistic, indicating the assembly quality and contiguity, was 3,904,904 bp with an L50 of 5. Assembly completeness was verified using BUSCO v5.4.7 (12).

**TABLE 1:** Summary statistics of the *Phlyctema vagabunda* strain 19EL15 genome

Features	Statistics
Assembly length (Mbp)	38.34
Number of scaffolds	13
Largest scaffold size (bp)	6,695,449
N50	3,904,904
N90	2,462,815
L50	5
L90	9
GC (%)	46.88
Predicted gene number	11,483
BUSCO completeness	93.8%
Complete and single-copy	93.5%
Complete and duplicated	0.3%
Fragmented	0.9%
Missing	5.27%

To annotate the genome, RNAseq data were obtained by growing strain 19EL15 on four media types—PDA, Sabouraud agar, apple leaf extract agar, and apple juice agar—for 1 week at 20°C. Mycelium samples were ground in liquid nitrogen and pooled. Total RNA was isolated using Plant/Fungi Total RNA Purification Kit (Norgen Biotek Corporation, Thorold, Canada). Libraries were prepared with the Novogene Stranded RNA Library Prep Set (PT044) designed for directional polyA libraries. Sequencing on Illumina NovoSeq 6000 (2 × 150 bp) produced 6,999,493 paired-end reads that were quality-checked with FastQC v0.11.9 and trimmed with Trimmomatic v0.33 (13). The genome is predicted to contain at least 11,483 protein-coding genes identified using the MAKER2 v3.01.02 pipeline (14), which incorporates a self-trained GeneMark-ES v4.10 model (15) and a *de novo* AUGUSTUS v3.3 model (16), both trained on the 19EL15 strain genome and transcriptomic data. Default parameters were used for all analyses, unless stated otherwise. Strain 19EL15 genome sequencing uncovers crucial genetic details to understand *P. vagabunda* biology.

## Data Availability

This Whole Genome Shotgun project has been deposited at DDBJ/ENA/GenBank under accession number JBFCZG000000000 (Bioproject: PRJNA1117780; Biosample: SAMN41579680; RNA reads: SRX27482699; ONT gDNA reads: SRX27482698; Illumina gDNA reads: SRX27482697).
